# InSAR analysis of surface deformation over permafrost to estimate active layer thickness based on one-dimensional heat transfer model of soils

**DOI:** 10.1038/srep15542

**Published:** 2015-10-20

**Authors:** Zhiwei Li, Rong Zhao, Jun Hu, Lianxing Wen, Guangcai Feng, Zeyu Zhang, Qijie Wang

**Affiliations:** 1 School of Geosciences and Info-Physics, Central South University, Changsha 410083, Hunan, China; 2 Department of Geosciences, State University of New York at Stony Brook, Stony Brook, NY 11794, USA; 3 Laboratory of Seismology and Physics of Earth’s Interior, School of Earth and Space Sciences, University of Science and Technology of China, Hefei, Anhui 230026, China

## Abstract

This paper presents a novel method to estimate active layer thickness (ALT) over permafrost based on InSAR (Interferometric Synthetic Aperture Radar) observation and the heat transfer model of soils. The time lags between the periodic feature of InSAR-observed surface deformation over permafrost and the meteorologically recorded temperatures are assumed to be the time intervals that the temperature maximum to diffuse from the ground surface downward to the bottom of the active layer. By exploiting the time lags and the one-dimensional heat transfer model of soils, we estimate the ALTs. Using the frozen soil region in southern Qinghai-Tibet Plateau (QTP) as examples, we provided a conceptual demonstration of the estimation of the InSAR pixel-wise ALTs. In the case study, the ALTs are ranging from 1.02 to 3.14 m and with an average of 1.95 m. The results are compatible with those sparse ALT observations/estimations by traditional methods, while with extraordinary high spatial resolution at pixel level (~40 meter). The presented method is simple, and can potentially be used for deriving high-resolution ALTs in other remote areas similar to QTP, where only sparse observations are available now.

The permafrost in Qinghai-Tibet Plateau (QTP), formed during the Quaternary, is extremely sensitive to climate changes and anthropogenic disturbances^1^,^2^,^3^. The active layer is the top soil layer above permafrost subject to seasonal freezing and thawing processes in response to air and surface temperature changes^4^. It plays a significant role in local landscape stability, carbon cycling, and socioeconomic development, and is one of keys to regulating the biological, hydrological, geophysical, and biogeochemical processes, because most exchanges of energy and mass fluxes between the surface and the atmosphere occur through it^4^,^5^,^6^,^7^,^8^,^27^. Active layer thickness (ALT), defined as the maximum soil thaw depth in the permafrost region in summer season^9^,^10^, is an crucial index of characterizing the permafrost degradation in QTP. It has been manifested that the thickness and extent of permafrost in QTP has been decreasing and the ALT has been increasing^3^,^7^,^11^.

Traditional methods for measuring ALT rely on point-based field surveys, such as drilling, soil temperature monitoring and ground-penetrating radar measurement^3^,^11^,^12^. Although characterized by high quality and good time continuity, these methods are extremely labor-intensive and time-consuming, and limited to only very sparse spots, due to the severe coldness and oxygen deficit environment in permafrost area like QTP. A few efforts have been made to estimate the ALTs in the whole QTP by extrapolating the *in-situ* measurements based on the Stefan and the Kudryavtsev’s formulas, which describe the empirical and/or statistical relationship between ALT and related factors such as vegetation type, elevation, air and ground temperature, etc. [*Pang et al*., 2009; *Zhang et al*., 2012; *Gangodagamage, C. et al*., 2014]^7^,^8^,^27^. Also a finite difference model for one-dimension heat conduction with phase change is used to investigate the ALTs in QTP based on the surface air temperature and snow depth^6^. However, these methods are region-based (implemented by interpolation/extrapolation) and with considerably low spatial resolution, generally a few to tens of kilometers.

InSAR has been widely used for monitoring the surface deformation caused by different geophysical processes, including the freezing and thawing process of the active layer in permafrost areas^13^,^14^,^15^,^16^,^17^,^18^,^19^,^20^. This makes it a very promising tool to map the changes of ALT. The first application of InSAR to invert ALT was done by *Liu et al*. [2012]^10^. They estimated the ALTs of the North Slope of Alaska from InSAR-derived surface subsidence of summer season. The work is however limited by the accuracy of the InSAR measurements, as obtained by using the SAR images of thaw season only, and the un-drained conditions of saturated and near-saturated soils. In this paper, we propose and test a new approach to estimate high-resolution ALTs based on InSAR observations. Its basic assumption is that the time lags between the InSAR-observed peak surface deformation and the meteorologically recorded maximum temperatures were equivalent to the time intervals that ground heat transfers from the top of the active layer to its bottom. By fully exploiting the time lags and the heat transfer model of soils, we estimate the ALTs. We demonstrate the concept of the ALT estimation using ENVISAT ASAR images over southern QTP.

## Study area

**Figure 1:**
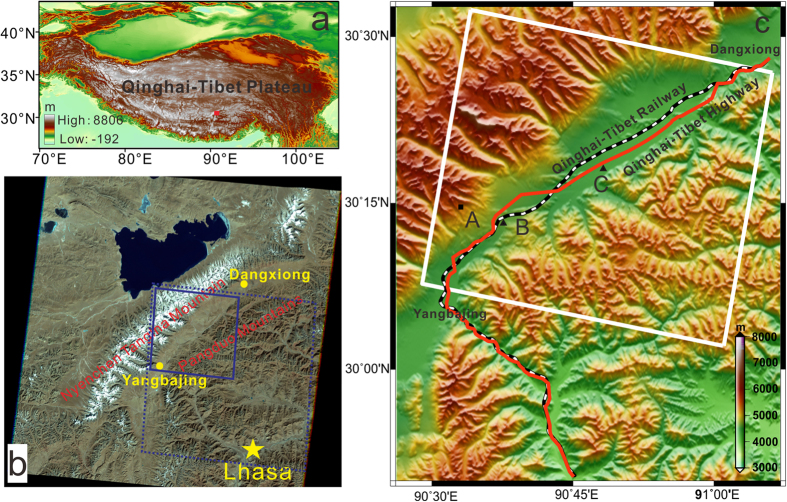
(**a**) The geographic map of QTP, produced with the 3-arcsecond SRTM DEM by using ARCGIS. The red square denotes the location of the study area. (**b**) The pseudo-color optical image of the study area, made from bands 4, 3, 2 images of Landsat 7 by using ENVI (http://earthexplorer.usgs.gov). The blue dashed and solid rectangle represents the spatial coverage of the selected ASAR images and the study area, respectively. The solid yellow circles and star indicate the locations of Dangxiong, Yangbajing and Lhasa, respectively. (**c**) Topographic relief map of the study area, generated with the 3-arcsecond SRTM DEM by using GMT software^28^. The solid black square (marked by A) refers to the starting point of phase unwrapping. The solid black triangles (marked by B and C) refer to the representative points lies along the QTR and the QTH, respectively.

The permafrost region covering Dangxiong to Yangbajing railway station in southern QTP (Fig. 1a) is chosen in this study. The surface deformation has been studied previously by InSAR and presents significant seasonal changes^14^. Figure 1b,c show the optical image of the study area and topographic relief map. The area lies between the Nyenchen Tanglha Mountain (NTM) and the Pangduo Mountain (PM). It is a wide tectonic basin (with the elevation of more than 4200 m), covered by the shrub grassland ecosystem, and approximately in parallel with the trending of the NTM, and belongs to the Dongya-Naqu rift system (Fig. 1b). The terrain in the whole region undulates roughly after the drastic uplift in the Quaternary (Fig. 1c). The average elevation of the NTM exceeds 6000 m above sea level (a. s. l.), with peak elevation of 7111 m, while that of the PM is only about 5200 m a. s. l. The Dangxiong-Yangbajing section of the Qinghai-Tibet Railway (QTR) and the Qinghai-Tibet highway (QTH) run through the area. A part of the Lhasa river flows through it.

The NTM is the boundary between the sub frigid and the temperate zone in the QTP^1^. Affected by the Indian monsoon and the NTM, the weather is cold and dry in winter, and warm, humid and with plenty of rain in summer. The study area has covered by the Quaternary deposits, and accumulated different sediments due to various factors (since Pliocene and early Pleistocene epoch) such as alluvial deposit, glaciers and ice water sediments, marsh peat sediments etc. As a result, the processes developed high terraces, wetlands and palaeo-alluvial-pluvial fans^21^. Therefore, the study area is underlain by different frozen soil^1^,^2^,^6^. In response to seasonal alternation, the ground surface in frozen soil areas undergoes dramatic freezing uplift and thawing settlement as much as 2–3 cm^14^, and the ALTs are increasingly thicker^3^,^7^,^11^.

## InSAR Observation

**Figure 2:**
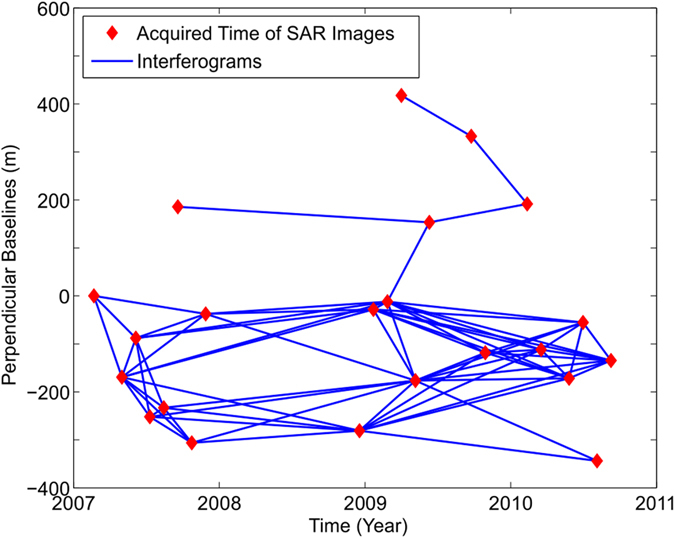
The temporal and spatial baselines of the formed SBAS interferograms.

We use the small baseline subset interferometry technique (SBAS-InSAR)^22^ and the ASAR imagery acquired by European Space Agency (ESA) ENVISAT satellite to investigate the surface deformation of the study area. In the analysis, a total of 22 descending ASAR images (track 405) spanning from 20 February 2007 to 7 September 2010 are included. The spatial coverage of the ASAR images is shown in Fig. 1b. These images were used to produce 68 interferograms with temporal baseline smaller than 700 days and spatial baseline smaller than 150 m, which can reduce the errors caused by the spatial and temporal decorrelation. The interferogram network shows that all the pairs are in one subset (Fig. 2). Standard two-pass D-InSAR method was applied to process the formed interferometric pairs and to generate differential interferograms^23^. In the processing, the 3-arcsecond SRTM digital elevation model (DEM) was used to remove the topographic phases, and the Delft precise orbits provided by ESA were adopted to reduce the orbit errors. To further suppress the noise, all the interferograms were multilooked by the factors of 2 and 10 along the range and azimuth directions, respectively. By setting the coherence threshold to 0.6, all the differential interferograms were unwrapped using the method of minimum cost flow (MCF)^24^, with respect to the same starting point (located on bare stable rock and marked by A in Fig. 1c).

As the ground surface underlain by frozen soils generally undergoes both long-term linear deformation and seasonal undulation, we adopted a superimposed model incorporating both deformation styles to approximate the low-pass (LP) temporal component of ground surface deformation^15^:


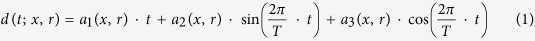


where *t* is the time interval with respect to the reference SAR image, *T* is the period of the seasonal undulations (assuming to be one year in this study), *α*_1_, *α*_2_ and *α*_3_ are the coefficients to be determined, and (*x, r*) represent the azimuth and range coordinates.

After replacing the linear deformation model in traditional SBAS-InSAR approach with Eq. (1), a weighted least squares inversion was carried out to estimate the coefficients *α*_1_, *α*_2_ and *α*_3_^15^. Then we calculated the residual interferograms by subtracting the modeled LP deformation from the original interferograms, and applied to them a spatial and temporal filtering to separate the possible atmospheric artifacts. Finally, a least squares estimation was employed to the atmosphere-corrected interferograms to generate the time series deformation corresponding to the 22 SAR acquisitions, using the SAR image acquired on 20 February 2007 as reference.

**Figure 3:**
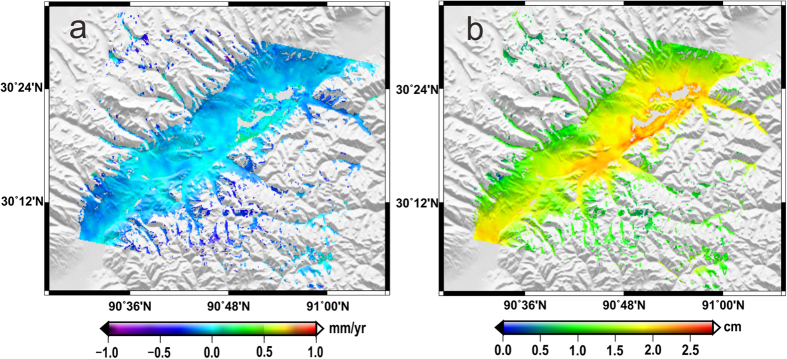
(**a**) The linear deformation velocity of the study area. (**b**) The amplitude of seasonal oscillation of the study area.

Figure 3 shows the linear deformation velocity (i.e., *α*_1_) and the amplitude (i.e., 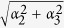) of seasonal oscillations of the study area over 2007–2010. As observed in Fig. 3a, the estimated linear deformation rates of most of the study area are from −1 to 0 mm/yr, which is possibly the result of secular permafrost thawing caused by temperature rise. Figure 3b shows the amplitude of the seasonal oscillations of the study area, ranging from 0.5 to 2.8 cm. The amplitudes of the areas along the QTR and the QTH were generally very large, due to the surface disturbance associated with engineering construction and carriage operation.

**Figure 4:**
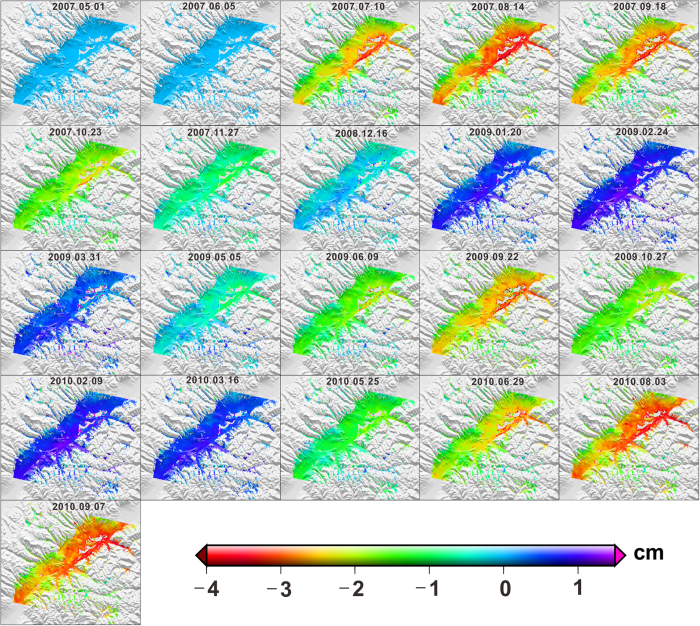
Time series deformation maps of the study area, with the observed date labeled in the top of each subplot.

The total time series deformation of the study area is presented in Fig. 4. It can be seen that the peak-to-peak seasonal oscillation within one year was up to 5.5 cm over 2007–2010. The surface deformation in the frozen soil area was characterized by a full disturbance cycle in each year, that is to say, thawing subsidence and freezing uplift occurred alternately. The land surface deformed significantly from July to September and from November to March of the following year. And the maximal seasonal settlement in summer and uplift in winter are nearly 4.0 and 1.5 cm, respectively, with reference to 20 February 2007, as noted earlier. We also observe that in cold season, the freezing uplift of the rifted basins in the southeast of NTM was larger than that of the area with high elevation. The reason is that these rifted basins are with rich soil water content (due to the moist sediments formed in paleo-environment), higher air temperature, and more anthropogenic activities, which can change the thermal balance between frozen soil and atmosphere layer and result in excess heat positive accumulation. The edge area of the rifted basins however was present with subsidence in a full year.

**Figure 5:**
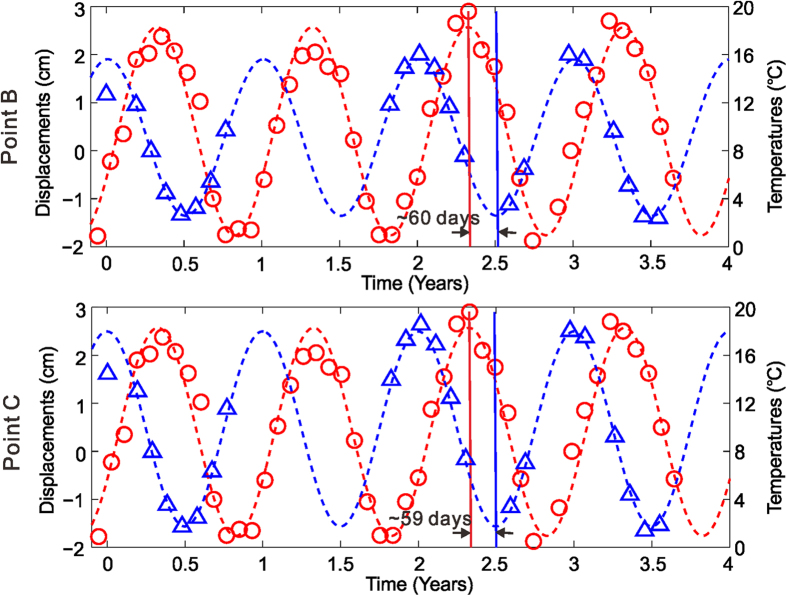
Comparison of the InSAR derived time series deformation (blue triangles) and the air temperature records of Lhasa station (red circles). The blue and red curves are fitted from the time series deformation and temperature, respectively, by periodic function. Points B and C are located along the QTR and QTH (Fig. 1c), respectively.

Figure 5 depicts the chronological sequence of the computed deformations (blue triangles) for the two points located along the QTR and QTH (marked by B and C in Fig. 1c), respectively. Also shown in Fig. 5 are the mean monthly air temperatures of Lhasa weather station (red circles). We can see that the air temperature of summer season in 2009 and 2010 was higher than that in 2007 and 2008, while for winter season the temperature was lower in 2009 and 2010 than in 2007 and 2008. The deformation and temperature sequences are then fitted by the sinusoidal function, shown as blue and red curves, respectively, in Fig. 5. The root mean square error (RMSE) of the temperature fitting are 1.43 °C and the correlation coefficient is as high as 0.97. The RMSEs of the deformation fitting of point B and C are 1.8 mm and 2.1 mm, respectively, and the correlation coefficient amounts to 0.99. These indicate the fitting are performed very well and the deformation/temperature exactly follows the periodic modal. Although both the air temperature and the time series deformation indicate the periodic patterns, the deformation is almost inversely correlated with the air temperature, because the higher the air temperature, the larger the settlement of the frozen soil (caused by the melting of the ice in the soil) will be, and vice versa. However, from the best-fitted blue and red curves, we can find that the periodic deformation and temperature are not totally out of phase. There exists a time lag of about two months between them, and we will fully exploit this time lag to estimate the ALT later.

## One-dimensional heat transfer model and ALT estimation

According to the heat conduction law, the temperature at soil depth *z* and time *t*, denoted as 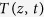, generally satisfies the following differential equation^25^


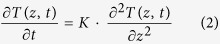


where *K* is the thermal diffusivity.

Commonly, the ground surface temperature in a year’s cycle presents the simple approximated periodic changes and is assumed to behave in accordance with^25^:


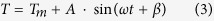


where *T* is the ground surface temperature, *T*_*m*_ is the average surface temperature, *A* is the amplitude of the surface temperature fluctuation, and *β* is the initial phase, 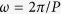, with the period being 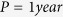 in this study.

By using the method of separation of variables to solve the partial differential Eq. (2) and considering Eq. (3), the temperature at soil depth *z* is obtained^25^:


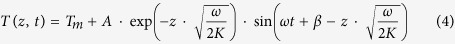


Eq. (4) is the general solution of one-dimensional heat transfer function of soils, under the condition that the effects of geothermal heat flux are ignored^25^. 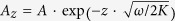 is the amplitude of the time-dependent soil temperature fluctuation, which decreases exponentially with soil depth. 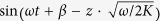 is the periodic part of the temperature at soil depth *z*. Comparing the periodic parts in Eqs (3) and (4), we find a phase difference 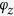 between the temperature fluctuations at ground surface and at soil depth *z*


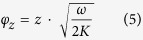


**Figure 6:**
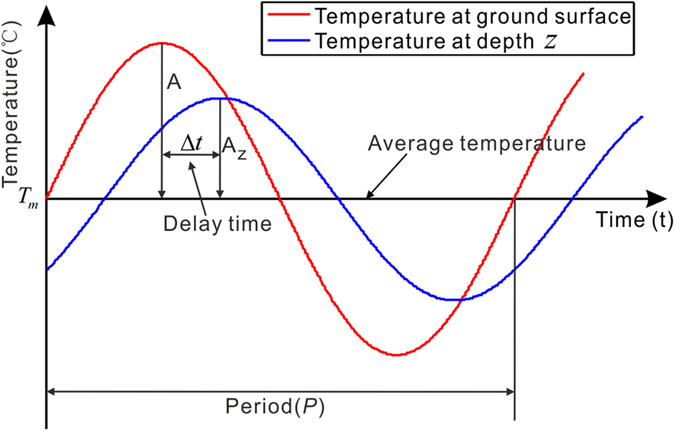
The sinusoidal variation of temperature at surface and at depth *z* below surface.

Generally, the temperature changes at ground surface and at soil depth *z* are not in phase^25^. Figure 6 shows the sinusoidal curves of the temperature at ground surface and at soil depth *z* for comparison. It is clear that the maximum temperature at soil depth *z* lags behind that at ground surface, namely, there exists a time delay Δ*t* between them. The time delay can be understood as the time required for the temperature maximum to diffuse from the ground surface downward to the soil depth *z*, which can be estimated by empirical method (discussed later). The time delay Δ*t* corresponds to a phase difference (i.e., the phase advance that the temperature at ground surface is over that at soil depth *z*)


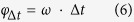


Both referring the phase differences between the temperature at ground surface and at soil depth *z*, 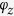 in Eq. (5) should be equal to 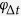 in Eq. (6), which yield


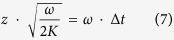


Rearranging Eq. (7), we get


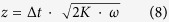


**Figure 7:**
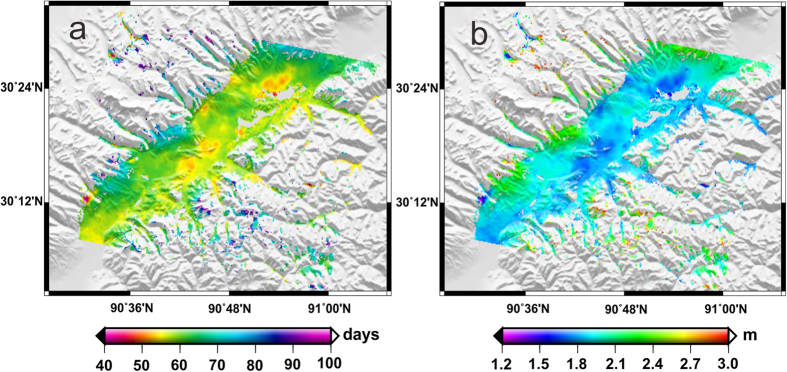
(**a**) Time lag map between the periodic curves of InSAR-observed thawing settlement and meteorologically recorded temperatures. (**b**) Estimated ALT map.

Eq. (8) can be exploited to derive the thawing depth of the frozen soil (i.e., ALT) if we know the time delay Δ*t* in one period of the temperature fluctuation (one year in the paper). As the ground surface temperature in the remote areas of QTP is generally lacking, we use air temperature in substitute. In addition, the temperature at soil thawing depth *z* is difficult to obtain. The time delay Δ*t* between the air temperature and the temperature at soil thawing depth *z* is still difficult to retrieve. We propose to use the thawing settlement to substitute the temperature at soil thawing depth *z* for the retrieving. The reasons are that (1) the thawing settlement increases with thawing depth (by assuming that surface subsidence is caused by the phase change of ice into water in the active layer), and (2) the thawing depth increases with the increase of soil temperature. Based on the above reasoning, the thawing settlement should then be almost linearly correlated with the soil temperature. We can simply assume that the time that the thawing settlement of the frozen soil reaches the maximum in a full cycle year is coincident with the time that the subsurface temperature reaches the maximum. Thus, the calculation of the time delay Δ*t* between the temperatures at ground surface and at maximum soil thawing depth *z* (ALT) reduces to analyze the time lag between the maximum air temperature and the maximum thawing settlement as measured by InSAR in a full cycle year. This analysis is implemented pixel by pixel and the time lag map for the study area is shown in Fig. 7a.

As shown in Fig. 7a, the time lags between the maximum air temperature and the maximum thawing settlement of most of the study area ranged from 39 to 98 days. In mountainous areas, the time lag was generally very long (e.g., up to 98 days), while in flat areas it was a bit short. The reason is that the permafrost in higher altitude areas are thicker than that in lower ones, which would generate a larger time lag, and additionally it needs more time to accumulate heat for melting frozen soil and counteract the influence of negative heat transferring upward. In general, the air temperature in QTP reached the maximum in middle July in each year, thus we can infer that the thawing depth in our study area reaches the maximum between the beginning of September and the middle of October, which was quite consistent with the known studies^6^.

The thermal diffusivity in Eq. (8) was derived from empirical values for specific soil given by *Zhang et al*. [2013]^26^. For simplicity, we adopted a same thermal diffusivity for the whole study area in this study by assuming the ground as a homogeneous body^7^. Then, along with the time lag Δ*t* in Fig. 7a, we derived the pixel-based maximum thawing depths (ALTs) of the study area (shown in Fig. 7b).

The averaged ALT of the entire study area (Fig. 7b) was 1.95 m over 2007–2010, and in the NTM and the PM area the ALT increased to about 3.14 m, while in the flatten basin with relatively low altitude it decreased to about 1.02 m. Along the QTR and QTH, the ALT was relatively uniform and with an average of less than 2.0 m, which was smaller than that of mountainous area. For the past few decades, the rising temperature has a greater impact on permafrost in mountainous than in flatten basin area. *Pang et al*. [2009]^7^ reported that the ALT around the QTH was greater than 2 m, with a spatial resolution of 0.5^°^ × 0.5^°^, as calculated by a climate-driven model based on the Kudryavtsev’s formula considering the effects of snow, vegetation and soil features. *Oelke and Zhang* [2007]^6^ estimated that the average ALT in sporadic and isolated permafrost regions of south-central Tibet was between 2–3 m, and the ALTs in the areas from Dangxiong to Lhasa railway station were about 2.2 ~ 3.4 m, with a resolution of 25 km × 25 km. Similarly, *Zhang et al*. [2012]^8^ calculated and predicted that the ALTs ranged from about 1.15 to 4.98 m, with an average of 2.61 m and a resolution of the 1 km × 1 km in the NTM and the Himalayan mountainous permafrost region by using the Stefan method and spatial interpolation. The ALTs estimated in this study (as shown in Fig. 7b) were quite compatible with those studies. However, its spatial resolution was improved to about 40 m (pixel size of the derived InSAR measurement), without any spatial interpolation. The high-resolution ALTs can more accurately characterize the spatial variations of the active layer in local areas, improve the permafrost and clod area ecosystem modeling, and provide valuable information for designing and planning of engineering structure, and possible prevention and mitigation of geohazard in QTP.

## Discussion and Conclusions

We present a new method to estimate the ALT based on InSAR observations and the one-dimensional heat transfer model of soils with a case study of Dangxiong County area at southern QTP as a concept demonstration. InSAR observations show that most of the study area underwent up to 5.5 cm peak-to-peak seasonal surface deformation in annual cycle. The time lags between the maximum thawing settlement and the highest air temperature in a full year were 39–98 days. These time lags are then translated into the ALTs through the heat transfer model of soils. The derived ALTs in the areas from Dangxiong to Yangbajing ranged from 1.02 to 3.14 m, with an average of 1.95 m. Compared with the sparse point-based filed measurements by traditional methods, the InSAR-based ALT estimates are of extraordinary high spatial resolution, about 40 m, and easily extended to cover large, remote and unreachable regions. Yet the estimation does not depend on a large number of datasets of ground surface temperature and soil water content, which are commonly unavailable over remote and depopulated regions of QTP. Remote sensing of ALTs using InSAR can raise our awareness on the variations of permafrost and the active layer, which will benefit the ecosystem research, designing and planning of engineering structure, and possible prevention and mitigation of geohazard in QTP.

However, there are rooms to refine the presented approach through continuing studies. Firstly, the introduction of sinusoidal model to SBAS-InSAR appears capable of approximating and modeling well the ground surface deformation of the frozen soil. However in reality, the freezing and thawing surface deformation is affected by many factors such as precipitation, temperature, water content, heat flow, and so on, which may not vary in accordance with the sinusoidal model. Secondly, the one-dimensional heat transfer formula is derived under the assumption of homogeneous ground, and single thermal diffusivity in the process of estimating the ALTs of the entire study area, without considering the differences of the frozen soil conditions. Actually, this practice is just too coarse because that the study area has several types of frozen soil and the soil properties are not the same throughout the active layer, which makes the thermal properties of the active layer inhomogeneous leading to bias to the estimated ALTs. In addition, the lack of *in-situ* measurements of surface subsidence and ALT prevent more detailed and quantitative evaluations of our results. Moreover, the constructed relationship between the time lag of the surface subsidence and the summer peak air temperature is an undesirable simplification in terms of lacking fully considering the actual physical process involved.

Nevertheless, we construct the relationship between the ALT and the time lag between periodic surface deformation over permafrost and air temperature, and demonstrate the capability of remote-sensing of ALTs from InSAR measurements and the one-dimensional heat transfer model of soils. This is the first attempt to estimate ALT by using the heat conductivity equation and InSAR observations. In the future, we will further develop new deformation model and use SAR data acquired from multiple satellites to detect surface deformation over permafrost, and construct more complicated inversion model (e.g., inhomogeneous, site-specific) to estimate ALTs over QTPs to fill the spatial gaps of ground-measurements.

## Additional Information

**How to cite this article**: Li, Z. *et al*. InSAR analysis of surface deformation over permafrost to estimate active layer thickness based on one-dimensional heat transfer model of soils. *Sci. Rep*. **5**, 15542; doi: 10.1038/srep15542 (2015).
